# Screening for visceral leishmaniasis in humans and animals in Laos

**DOI:** 10.1186/s41182-025-00782-w

**Published:** 2025-08-01

**Authors:** Tamalee Roberts, Anousone Douangnouvong, Matthew T. Robinson, Koukeo Phommasone, Saykham Phaxayaseng, Valy Keoluangkhot, Khamsing Vongphayloth, Aphaphone Adsamoud, Othila Rasphone, Leeyounjera Yang, Phonelevanh Phoumin, Manivone Simmalavong, Peter Christensen, Tom Hughes, Adisone Temmerath, Alex Inthavong, Phoummavanh Inthapanya, Sivone Punyasith, Phouvong Phommachanh, Wattana Theppangna, Syseng Khounsy, Susath Vongphachanh, Stuart D. Blacksell, Paul N. Newton, Mayfong Mayxay, Elizabeth A. Ashley

**Affiliations:** 1 Lao-Oxford-Mahosot Hospital-Wellcome Trust Research Unit, Microbiology Laboratory, Mahosot Hospital, Mahosot Road, Vientiane, Lao PDR; 2 Centre for Tropical Medicine and Global Health, Nuffield Department of Medicine, University of Oxford, Oxford, UK; 3 HIV Unit, Setthathirath Hospital, Vientiane, Lao PDR; 4 Department of Infectious Diseases, Mahosot Hospital, Vientiane, Lao PDR; 5 Institut Pasteur du Laos, Laboratory of Vector-Borne Diseases, Vientiane, Lao PDR; 6 Mahidol-Oxford Tropical Medicine Research Unit, Faculty of Tropical Medicine, Mahidol University, Bangkok, Thailand; 7 Conservation Medicine, Sungai Buloh, Selangor, Malaysia; 8 National Animal Health Laboratory, Vientiane, Lao PDR; 9 Mahosot Hospital, Vientiane, Lao PDR; 10 Institute for Research and Education Development, University of Health Sciences, Vientiane, Lao PDR; 11 Saw Swee Hock School of Public Health, National University of Singapore, Singapore, Singapore

**Keywords:** Visceral leishmaniasis, *Leishmania*, Lao PDR, Ruminants, Zoonosis

## Abstract

**Background:**

Visceral leishmaniasis (VL) is a vector-borne protozoan disease with a global distribution, with higher rates of infection associated with HIV. Zoonotic species of *Leishmania* have also been reported infecting domestic animals. Reports of VL are increasing in Southeast Asia, with over 200 cases reported in Thailand since the first autochthonous case in 1999, and recently the first patients have been reported from Vietnam and Cambodia. However, no cases of VL have been reported from Lao PDR (Laos) and clinical awareness of the disease is limited. This study aimed to investigate whether *Leishmania* is circulating in Laos by screening people living with HIV, stored samples from unselected patients with fever, and ruminants taken to abattoirs.

**Methods:**

People living with HIV from two specialist units in Vientiane Capital had EDTA blood taken and DNA extracted and tested for *Leishmania* by nested-PCR. Stored serum samples from patients presenting to Mahosot Hospital with fever and without known HIV infection, as well as serum from goats, cows and buffalo taken to abattoirs in four provinces in Laos were tested for *Leishmania* using the InBios Kalazar *Detect* Rapid Test.

**Results:**

There were 1015 people living with HIV tested between 2021 and 2024 for *Leishmania* by nested-PCR, all of whom were negative. Of 511 human serum samples collected between 2005 and 2023, two (0.4%) tested positive by rapid test. These samples were identified as coming from the same patient, with samples taken 10 months apart. There were 5/159 (3.1%) ruminant serum samples positive by rapid test with 3/45 (6.7%) buffalo positive, 2/47 (4.3%) goat positive and 0/67 cows positive.

**Conclusions:**

This study suggests *Leishmania* may be circulating in Laos with undetected cases. Further investigation is needed to confirm the findings, determine at-risk populations and increase clinical awareness of the disease. This study expands on the current regional knowledge on leishmaniasis and shows the need for further epidemiological studies.

**Supplementary Information:**

The online version contains supplementary material available at 10.1186/s41182-025-00782-w.

## Background

Leishmaniasis is a vector-borne neglected tropical disease caused by protozoa of the genus *Leishmania* with the parasite most commonly transmitted via the bite of phlebotomine sand flies [1]. There are three different clinical syndromes: cutaneous leishmaniasis (CL) manifests as ulcerated skin lesions and papulo-nodular lesions; mucocutaneous leishmaniasis (MCL) affects the mucous membranes; and visceral leishmaniasis (VL) is a systemic, potentially lethal disease that has emerged as an opportunistic infection associated with HIV [2]. There are an estimated 600,000 to 1 million new cases of CL and 50,000–90,000 cases of VL reported annually worldwide [3]. Concomitant infection with HIV increases the risk of developing active VL, and HIV–*Leishmania* coinfection is a significant problem for public health with severe morbidity and limited treatment options for infected people [2]. Without antiretroviral therapy, VL is invariably fatal in patients living with HIV. Leishmaniasis is one of the most neglected tropical diseases which predominantly affects the poor and is more common in rural areas of tropical and subtropical regions [4]. There is potential for *Leishmania* infections to be misdiagnosed as other diseases, such as fungal infections or tuberculosis, resulting in incorrect treatment. There are several zoonotic species of *Leishmania* in domestic animal reservoirs, including dogs, cats, cattle and goats [5, 6]. With changing climates and increased temperatures, *Leishmania* vectors are predicted to expand to new areas, increasing the transmission range [7, 8].

Methods for the detection of *Leishmania* include immunochromatographic tests (ICT), latex agglutination, microscopy of stained sample smears and PCR [9]. Rapid diagnostic tests (RDTs) such as ICTs have the potential to be used in the field for rapid diagnosis of VL without the need for highly trained staff and equipment. Several ICTs are available for the detection of *Leishmania* using the recombinant test antigen rK39. However, ICTs have been shown to have higher sensitivity and specificity in the Indian subcontinent compared to North Africa and the Mediterranean [10]. This may be due to the different endemic species circulating in the different areas, but it may also be due to the parasite load found in patients tested. In Thailand, one study evaluated the Kalazar *Detect* Rapid Test ICT (InBios) on 10 patient samples with known *L. martiniquensis* infection, and all were negative [11]. A case report in a non-HIV infected Thai child also showed the lack of ability for the Kalazar *Detect* Rapid Test ICT to detect VL with *L. martiniquensis* [12]. It is also not know if the ICT cross reacts with other organisms. However, no other ICTs have been developed specifically for Southeast Asian strains. A limitation of ICTs is that people with advanced HIV infection frequently have low or undetectable anti-Leishmanial antibodies and may give a false negative result [13]. Patient HIV status should be taken into account when choosing the appropriate diagnostic test.

In Thailand, the first autochthonous VL case was described from a southern province (Surat Thani) in a 3-year-old girl in 1999 [14]. Since this report, there have been over 200 reports of locally acquired symptomatic and asymptomatic leishmaniasis with reports predominately from southern and northern Thailand [15–18]. The number of VL–HIV coinfection cases are increasing in Thailand with *L. (Mundinia) martiniquensis* the most common reported species in Thailand in both immunocompetent and immunosuppressed people, and *L. (Mundinia) orientalis* which is rarer and has only been reported from Thailand and is primarily the cause of CL and concomitant VL in immunosuppressed patients [19]. A VL–HIV coinfection was also reported in Vietnam in 2018 [20], and the first VL patient in Cambodia was recently described [21]. There have also been occasional reports of VL in individuals from Myanmar [22, 23].

Lao PDR (Laos) is a land-linked lower- middle-income country in Southeast Asia. There are approximately 7 million people with 67% living in rural areas [24]. The Mekong River acts as a natural border separating the majority of Laos and Thailand, however, for the two northern provinces of Bokeo and Xayabury in Laos, the Mekong River passes through Laos resulting in land borders with Chiang Rai, Phayao and Nan Provinces in Thailand. The terrain and climate are similar on both sides of the border and, with no physical barriers, *Leishmania* vectors could easily disseminate between countries. While there have been numerous reports of leishmaniasis from northern Thailand, no cases have been reported from Laos, though awareness of the disease is limited. There was an investigation into *Leishmania* in Laos reported in the Bulletin de la Société Médico 1937, which screened dog spleen samples for *Leishmania* by microscopy in Vientiane Capital, and all samples were negative. A recent study screened 1,464 sand flies in 227 pools from caves in northern Laos by nested-PCR for *Leishmania*, but all were negative (submitted), though caves were not generally located near villages. No imported cases of leishmaniasis have been reported in the country. There were approximately 20,000 people living with HIV in Laos in 2023, with a prevalence in adults of 0.4% [25].

Due to the increasing number of *Leishmania* cases seen in northern Thailand and the lack of data from Laos, the aim of the study was to investigate the presence of *Leishmania* in Laos. This was carried out by screening newly diagnosed patients with advanced HIV infection by PCR, as they are considered a high-risk group for VL, and screening by ICT on stored serum specimens from patients with no known history of HIV, and abattoir ruminants and goats. PCR was carried out on blood from people living with HIV due to ICTs potentially giving false negative results for HIV-positive samples. Serum from patients with no known history of HIV and animal samples were tested by ICT due to a lack of buffy coat sample availability for PCR, to evaluate the ICT to test the usefulness of the test for testing in areas without PCR equipment.

## Materials and methods

### People living with HIV

Newly diagnosed individuals with HIV, or patients with HIV who had not had treatment for more than 3 months and who were aged >15 years and presented to Setthathirath Hospital HIV Unit or Mahosot Hospital Infectious Diseases ward in Vientiane Capital were included. Setthathirath Hospital HIV Unit is the national referral centre for newly diagnosed HIV patients with a bed capacity of 18, with 323 and 369 patients admitted in 2022 and 2023, respectively, and seeing approximately 14,500 HIV out-patients a year. Mahosot Hospital Infectious Diseases ward has a bed capacity of 32, with approximately 7,000 HIV out-patients seen yearly in 2022 and 2023.

After obtaining written informed consent, 3–5 ml of blood in EDTA was collected and sent to Mahosot Hospital Microbiology Laboratory where it was spun (3200 rpm for 8 min) and the buffy coat and plasma were separated. DNA was extracted from the buffy coat using the GeneJET Genomic DNA Purification Kit (ThermoFisher Scientific, UK). A nested-PCR was performed on extracted DNA, amplifying a 300–350 bp fragment of the internal transcribed spacer 1 (ITS1) region of the rRNA gene using primers LITSR and L5.8S for the primary PCR reaction and LITS2R and L5.8S inner for the secondary PCR reaction (Table S1) [26, 27]. PCR mixtures were 25 µl and consisted of 0.5 µM each primer, 0.2 mM each dNTP, 2 mM MgCl2, 1× PCR buffer, 1 U Platinum Taq DNA polymerase (Invitrogen, Carlsbad CA), and either 2 µL DNA or 4 µL first round PCR product. Thermal cycling consisted of 2 min at 94 °C, followed by 35 cycles of 94 °C at 20 s, 53 °C for 30 s and 72 °C for 1 min, with a final extension step of 72 °C for 6 min. PCR products were run on a 1.5% agarose gel electrophoresis prepared with 1 X Tris–borate–EDTA buffer at 100 V for approximately 1 h. PCR-positive products were purified using GeneJET Gel Extraction Kit (ThermoFisher Scientific, UK) and sent for sequencing at a commercial facility (MACROGEN, South Korea). The PCR was validated with four positive controls (*L. orientalis, L martiniquensis, L. tropica* and *L. major*) and a negative control, and two positives and a negative control were used for all PCR runs.

### Patients with fever and no known history of HIV

Selected stored serum samples from patients aged >15 years with undiagnosed fever as well as anaemia or abdominal pain with a wide home geographical distribution presenting to Mahosot Hospital were screened for *Leishmania* using the InBios Kalazar *Detect* Rapid Test ICT following the manufacturer’s instructions. Stored serum samples were collected as part of a long-term study on the causes of fever in Laos. Samples from each province were selected to cover the geographical range. The InBios Kalazar *Detect* Rapid Test is a qualitative test for the detection of IgG antibodies to members of the *L. donovani* complex using the recombinant test antigen rK39.

Patient information including age, sex, home location, history of travel, recent contact with animals, occupation, clinical presentation, temperature, underlying illness, CBC and CD4 count were collected where available.

### Animals from abattoirs

A selection of serum from buffalo, cows and goats taken to abattoirs from four provinces in Laos, three bordering Thailand (Bokeo, Xayabury, and Vientiane Province) and one bordering Cambodia and Vietnam (Attapeu), were selected. These provinces were selected as they border regions of countries where cases of *Leishmania* have previously been found. Animals varied in age and sex. Samples were collected as part of the US-funded Defense Threat Reduction Agency (DTRA) programme that studied the surveillance of transboundary and One Health pathogens, gathering samples from 18 provincial abattoir sites in Laos. Animals came from smallholder and household farms. Serum samples were tested at the Veterinary One-Health Reference Laboratory, at the National Animal Health Laboratory in Vientiane using the Kalazar *Detect* Rapid Test ICT following the manufacturer’s instructions.

### Clinician engagement

As leishmaniasis has never been diagnosed in Laos before, most clinicians were not aware of the disease before the project, so clinical trainings on the disease and the study were carried out at the two study units, which are also the main infectious diseases and HIV units in the country. These clinical training sessions described the disease, symptoms, transmission, diagnosis and treatment. There was continual clinician engagement throughout the project.

### Data analysis

Data were stored in Microsoft Excel and Access databases. Descriptive analysis was completed using Microsoft Excel. Maps were created using QGIS version 3.32.2-Lima.

## Results

### People living with HIV

EDTA blood samples were collected from 1015 patients presenting to the two sites between May 2021 and January 2024. There were 417 samples from the Mahosot Hospital Infectious Diseases ward and 598 from the Setthathirath Hospital HIV Unit. The median age was 29 years (range 16–72 years), with 287/1015 female (28.3%). Patients came from 16/18 provinces in Laos, with the majority from Vientiane Capital, the main catchment area for these hospitals (792 patients, 78.0%) (S2 Table, S1 Figure). CD4 count was available for 434 patients (42.8%) with the median CD4 count 229 cells/µl (range <50–940). All samples tested negative by nested-PCR for *Leishmania* (Table 1).

**Table 1:** Summary of results for patients with HIV tested by nested-PCR, patients with fever and no history of HIV and animals from abattoirs tested by InBios Kalazar *Detect* Rapid Test (ICT)

Sample type	Total	Male *n* (%)	Female *n* (%)	Positive by nested-PCR *n* (%)	Positive by ICT *n* (%)
People living with HIV	1015	728 (71.7)	287 (28.3)	0	NT
Patients with fever and no known history of HIV	511	300 (58.7)	211 (41.3)	1/2*	2 (0.4)
Buffalo	45	22 (48.9)	23 (51.1)	NT	3 (6.7)
Cow	67	40 (59.7)	27 (40.3)	NT	0
Goat	47	14 (29.8)	33 (70.2)	NT	2 (4.3)

*NT* not tested

^*^only the two ICT-positive patient samples were tested by PCR. Negative patients were not tested. One of the two ICT-positive samples gave a weak PCR band

### Patients with fever and no known history of HIV

A total of 511 stored serum samples from patients with no known history of HIV collected between January 2005 and October 2023 were tested using the Kalazar *Detect* Rapid Test ICT. The median patient age was 43 years (range 16–90 years), and there were 211 females (41.3%). Patients came from all 18 provinces, with the majority from Vientiane Capital (277, 54.2%) (Table S2, S1 Figure). Two serum samples (0.4%) were positive by ICT (Table 1). On further inspection, it was found that the two samples came from the same patient, with one sample collected in August 2014 and the other in June 2015. The patient was an 83-year-old female with prolonged fever, anaemia (WBC 16 × 10^3^ µL, platelets 542 × 10^3^ µL and Hct 10.8%) and abdominal tenderness. Blood cultures were negative and TB and HIV status was not known. The patient came from Vientiane Capital. The stored buffy coat from the patient was tested by PCR and gave a weak positive band but unfortunately there was not enough PCR product for sequencing (S1 Figure).

### Animals from abattoirs

A total of 2/47 (4.3%) goat serum samples collected between March and May 2017, 3/45 (6.7%) buffalo and 0/67 cow samples collected between October 2021 and June 2023 were positive for *Leishmania* by the Kalazar *Detect* Rapid Test ICT (Table 1). Four positive samples came from provinces that border northern Thailand: two goat samples from Xayabury Province and two buffalo samples from Vientiane Province. Additionally, another positive buffalo sample came from Attapeu Province which borders Northern Cambodia and Central Vietnam (Table S3, S2 Figure). Goats ranged in age from 7 months to 4 years, with the two positives from goats aged 7–9 months. Buffalo ages ranged from 2–8 years, with two 3-year-olds and a 7-year-old positive. Cows ranged from 1–18 years old. No sickness was noted for any animals.

## Discussion

This is the first prevalence study on visceral leishmaniasis in Laos. One patient and five animals tested positive for *Leishmania* antibodies by ICT, and no people living with HIV tested positive by PCR. Due to the presence of circulating *Leishmania* in Thailand, and in particular provinces in northern Thailand that border Laos, it is surprising that there was not higher prevalence of the disease found in Laos. A recent study on sand flies from northern Laos also did not detect any *Leishmania* by nested-PCR, though sample numbers were limited and not from within villages (submitted). Unfortunately, due to SARS-CoV-2 travel restrictions during the study period, we were not able to focus on areas in Laos that border provinces in Thailand where a high prevalence of the disease has been found, although most patients diagnosed with HIV in the country are referred to the central level to enable them to be established on to treatment. However, this is the first report of *Leishmania* in Laos and shows the need for further studies.

During the study period, the first case of leishmaniasis in Cambodia was diagnosed [21], and several more cases were reported from Chiang Rai Province, including disseminated cutaneous, mucocutaneous and VL diagnosed in a patient with HIV [15, 28], and cutaneous leishmaniasis in an infant from southern Thailand [29]. *Leishmania* was also detected in *Culicoides* biting midges from a cave and communities with previously diagnosed leishmaniasis in Chiang Rai and Lampang Provinces in Northern Thailand, and from the vicinity of *Leishmania* patients in southern Thailand [30–32]. All these studies show that leishmaniasis is now an endemic disease in Southeast Asia but the true prevalence is still unknown. This also shows the importance of clinical awareness of the disease as it continues to be found in more areas in Southeast Asia. At the start of the study there was little awareness of leishmaniasis among Lao clinicians and most people were not aware of what a sand fly was (Heen foy sai , in local language). Through clinical trainings for the project and dissemination of results, there is now a wider knowledge of the disease and clinicians have started requesting samples to be tested. Liposomal amphotericin B is approved as a treatment by the American Food and Drug Administration for VL and miltefosine for VL caused specifically by *L. donovani,* but both these medicines are not available in Laos with only amphotericin B deoxycholate currently available for treatment [33]. If cases of VL were detected in the country, procurement of medicines from neighbouring countries would be necessary. In Thailand, a detectable HIV viral load >50 copies/mL, CD4+ levels 200–500 cells/µL, and living in stilt houses were associated with *Leishmania* infection [16]. The majority of patients with HIV in the current study had low CD4 counts (median 229 cells/µl) and though the viral load was not known, patients were either newly diagnosed or had not been on medication for >3 months. Although stilted houses are common in rural areas in Laos this question was not included in the clinical history. Asymptomatic infection may also play a role in transmission in Thailand, particularly in northern Thailand, and this could also occur in Laos.

There were two goat and three buffalo samples positive by ICT for *Leishmania* in this study, though they were not able to be confirmed by testing by PCR to determine which species they were or if they were false positives. The ICT has not been validated for animals and may be cross-reacting with other common organisms found in animals. In Bangladesh 4.74% of cattle and goat samples were positive using the same ICT as in this study but were negative by PCR [34]. In Nepal, a study found that 5–16% of cows, goats and buffaloes were positive for *Leishmania* by PCR [35]. One buffalo-positive sample in our study came from Attapeu Province, which borders Stung Treng Province in northern Cambodia, where the first case of *Leishmania* in Cambodia was diagnosed [21] and the other positive samples came from provinces bordering northern Thailand where leishmaniasis is endemic. In Laos, animals and humans live in close communities and may have a lot of contact. Studying livestock and pets will be important for determining transmission dynamics for *Leishmania* in Laos and further studies including confirmation with PCR are needed.

The spread of *Leishmania* has been linked to environmental changes such as deforestation, building dams and urbanisation, as well as climate change, with vectors increasing in range with increased temperatures. What impact environmental and climate change will have on vectors for *Leishmania* in Laos and the region is unknown, but we may see an increase in cases and spread of the disease into new areas. *Leishmania* elimination programmes are proving successful in South Asia, particularly in Bangladesh, where VL has been classified as eliminated, the first country to do so [36]. *Leishmania* elimination programmes in the region may affect the spread of *Leishmania* into Southeast Asia with new foci of transmission emerging in non-endemic areas. This area needs to be monitored, especially in Myanmar and Laos where knowledge of the disease is limited and there are no surveillance programmes.

There were several limitations to this study. First, the study started during the SARS-CoV-2 pandemic, and the original plan to collect samples from across Laos was not possible due to lockdowns and travel restrictions. Therefore, the study focused on patients presenting to the two main HIV units in Vientiane Capital. This means that a wide geographical collection was not possible, and there were limited numbers of patients from border areas with northern Thailand, which is where we could have expected some cases. A larger study with collection from humans, animals and vectors in northern Laos is required to thoroughly investigate and determine the true prevalence of leishmaniasis in Laos. A study is underway investigating cutaneous leishmaniasis in Laos, which will also help complete the picture. Another limitation of the study was that the CD4 machines in all centres ran out of reagents in mid-2023 and no replacements were received during the rest of the study period. This means that we do not have HIV staging information for all patients. Unfortunately, we were not able to confirm any of the ICT-positive samples by PCR and therefore, were not able to validate the test. This could mean that they are false positives, but also that there might be false negatives with the test not being able to detect *L. martiniquensis*. Due to the positive patient sample having been collected 10 years ago, we were not able to contact the patient to find out about travel history or other underlying diseases which may cause a cross-reaction with the ICT. Apart from abdominal tenderness, there were no other clinical signs specific to *Leishmania.* While the PCR for this patient sample also gave a weak positive, without further testing, we cannot confirm this as a true *Leishmania* case. While the ICT proved easy to use, a full evaluation of its sensitivity and specificity for detecting regional species would be required before deploying to the field. Two previous reports from Thailand showed the Kalazar *Detect* Rapid Test was unable to detect a *L. martiniquensis* cases [11, 12] but there have been no other reports of the test used in Southeast Asia. While the ITS1 has been widely used for *Leishmania* detection, the use of other markers in future studies may be useful to ensure positive cases are not being missed.

## Conclusion

This is the first investigation of leishmaniasis in humans in Laos and results from humans and animals shows limited evidence for the disease circulating in the country. Further studies are required to determine the true prevalence and high-risk areas within the country with vector studies expanded to include trapping sand flies in villages and investigating other potential vectors such as biting midges.

## Supplementary Information

Additional file 1.

## Data Availability

The metadata supporting the conclusions of this article with de-identified data, and the supporting information files are available in the Figshare repository 10.6084/m9.figshare.28935005.
