# Supplementary material for: Screening for visceral leishmaniasis in humans and animals in Laos
Source: Trop Med Health. 2025 Aug 1;53:101. doi: 10.1186/s41182-025-00782-w (PMC12315420; doi:10.1186/s41182-025-00782-w)
Supplement: Supplementary file 1 — Additional file 1. [file 41182_2025_782_MOESM1_ESM.docx]

**Screening for visceral leishmaniasis in humans and animals in Laos**

Tamalee Roberts^1,2*^, Anousone Douangnouvong^1^, Matthew T Robinson^1,2^, Koukeo Phommasone^1^, Saykham Phaxayaseng^3^, Valy Keoluangkhot^4^, Khamsing Vongphayloth^5^, Aphaphone Adsamoud^1^, Othila Rasphone^4^, Leeyounjera Yang^4^, Phonelevanh Phoumin^1^, Manivone Simmalavong^1^, Peter Christensen^6^, Tom Hughes^6,7^, Adisone Temmerath^6,8^, Alex Inthavong^6,8^, Phoummavanh Inthapanya^6,8^, Sivone Punyasith^6,8^, Phouvong Phommachanh^8^, Wattana Theppangna^8^, Syseng Khounsy^6,8^, Susath Vongphachanh^9^, Stuart D Blacksell^2,6^, Paul N Newton^1,2,6^, Mayfong Mayxay^1,2,10,11^, Elizabeth A Ashley^1,2^

^1^Lao-Oxford-Mahosot Hospital-Wellcome Trust Research Unit, Microbiology Laboratory, Mahosot Hospital, Vientiane, Lao PDR

^2^Centre for Tropical Medicine and Global Health, Nuffield Department of Medicine, University of Oxford, Oxford, United Kingdom

^3^HIV Unit, Setthathirath Hospital, Vientiane, Lao PDR

^4^ Department of Infectious Diseases, Mahosot Hospital, Vientiane, Lao PDR

^5^Institut Pasteur du Laos, Laboratory of Vector-Borne Diseases, Vientiane, Lao PDR

^6^Mahidol-Oxford Tropical Medicine Research Unit, Faculty of Tropical Medicine, Mahidol University, Bangkok, Thailand

^7^Conservation Medicine, Sungai Buloh, Selangor, Malaysia

^8^National Animal Health Laboratory, Vientiane, Lao PDR

^9^Mahosot Hospital, Vientiane, Lao PDR

^10^Institute for Research and Education Development, University of Health Sciences, Vientiane, Lao PDR

^11^Saw Swee Hock School of Public Health, National University of Singapore, Singapore

**Supporting information**

**Table S1**. Primers used for *Leishmania* nested- PCR of the ITS1 region

| Round | Primer | Sequence (5’-3’) | Product size (bp) |
| --- | --- | --- | --- |
| 1 | LITSR | CTGGATCATTTTCCGATG | 300-350 |
|  | L5.8S | TGATACCACTTATCGCACTT |  |
| 2 | LITS2R | CTGGATCATTTTCCGATGATT | 300-350 |
|  | L5.8S inner | GTTATGTGAGCCGTTATCC |  |

**Table S2**. Patient home province for patients living with HIV tested by nested- PCR and patients with fever and no known history of HIV tested by Kalazar *Detect* Rapid Test (ICT)

| Province | Number of patients living with HIV tested by PCR by province (%) | Number of patients with fever and no known history of HIV tested by ICT by Province (%) |
| --- | --- | --- |
| Attapeu | 1 (0.1) | 4 (0.8) |
| Bokeo | 1 (0.1) | 2 (0.4) |
| Bolikhamxay | 39 (3.8) | 23 (4.5) |
| Champasak | 1 (0.1) | 8 (1.6) |
| Huaphan | 2 (0.2) | 10 (2.0) |
| Khammuane | 1 (0.1) | 7 (1.4) |
| Luang Namtha | 2 (0.2) | 9 (1.8) |
| Luang Prabang | 12 (1.2) | 20 (3.9) |
| Oudomxay | 4 (0.4) | 9 (1.8) |
| Phongsaly | 2 (0.2) | 2 (0.4) |
| Savannakhet | 2 (0.2) | 6 (1.2) |
| Salavan | 0 | 5 (1.0) |
| Sekong | 0 | 4 (0.8) |
| Vientiane Capital | 792 (78.0) | 277 (54.2) |
| Vientiane Province | 89 (8.8) | 81 (18.9) |
| Xayabury | 8 (0.8) | 19 (3.7) |
| Xaysomboun | 7 (0.7) | 6 (1.2) |
| Xieng Khuang | 30 (3.0) | 19 (3.7) |
| Unknown | 22 (2.2) | 0 |
| Total | 1015 | 511 |

**Table S3.** Animal origin by province and the number tested positive by Kalazar *Detect* Rapid Test (ICT)

| **Province** | **No. of buffalo samples (no. positive, %)** | **No. of cow samples (no. positive, %)** | **No. of goat samples (no. positive, %)** |
| --- | --- | --- | --- |
| Bokeo | 14 (0) | 14 (0) | 0 |
| Xayabury | 14 (0) | 14 (0) | 33 (2, 6.0) |
| Vientiane Province | 3 (2, 6.7) | 25 (0) | 0 |
| Attapeu | 14 (1, 7.1) | 14 (0) | 14 (0) |


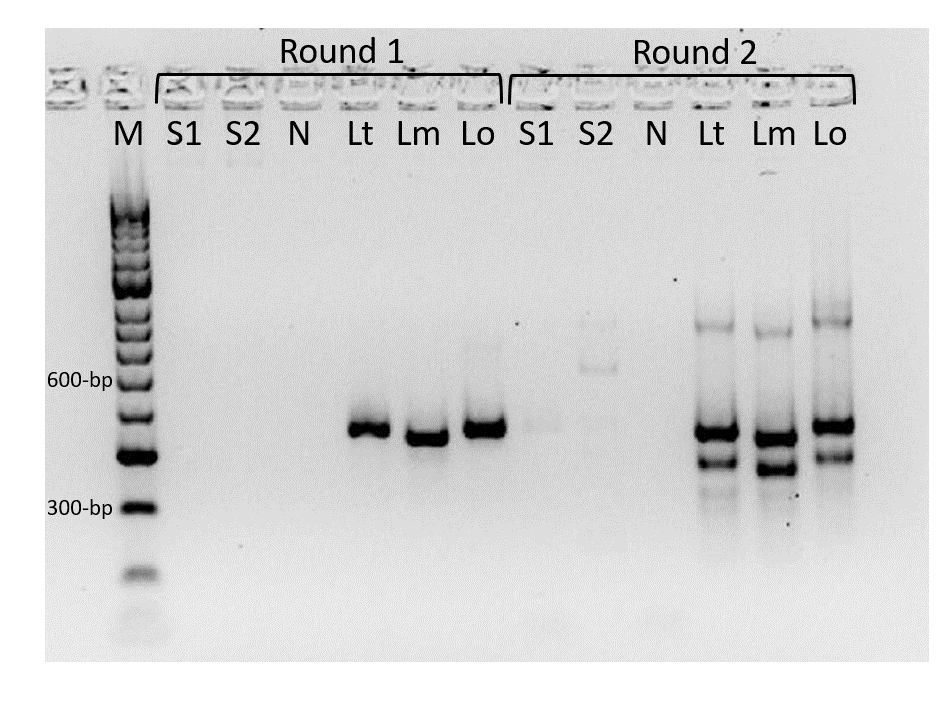


**Figure S1**. Gel picture from nested PCR of the ITS1 region of the rRNA gene. S1= the first sample from the patient which was ICT positive with the sample taken in 2014. S2= second sample from the same patient which was ICT positive with the sample taken in 2015. N= Negative control. Lt= *L. tropica* control. Lm= *L. major.* Lo= *L. orientalis*


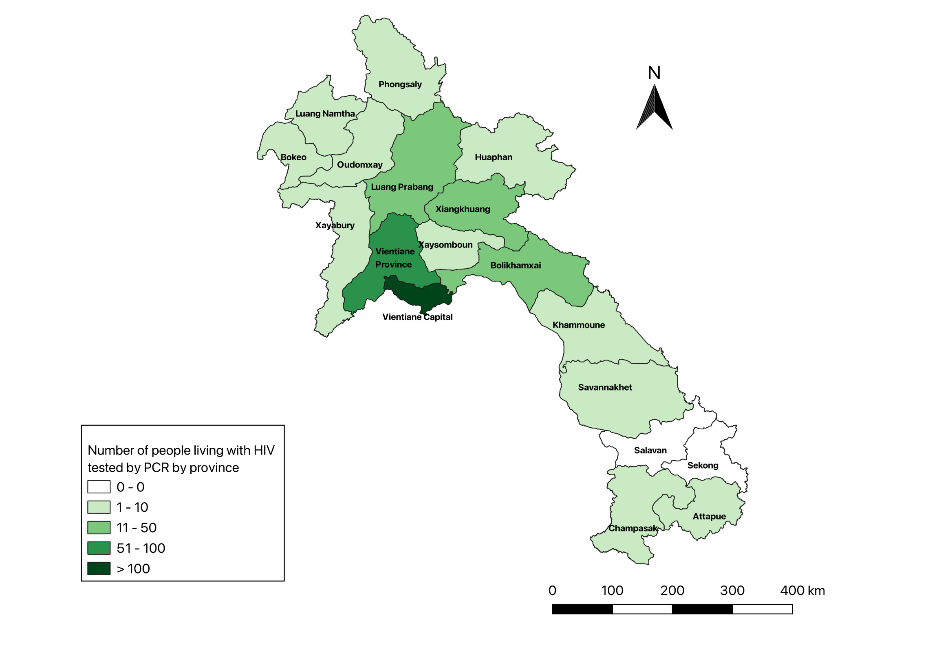

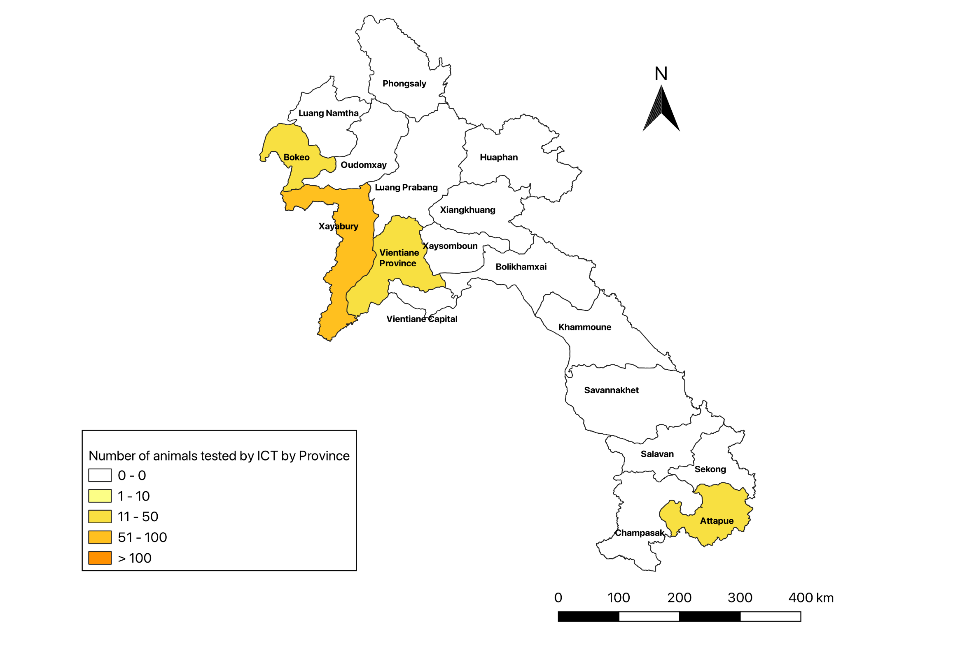

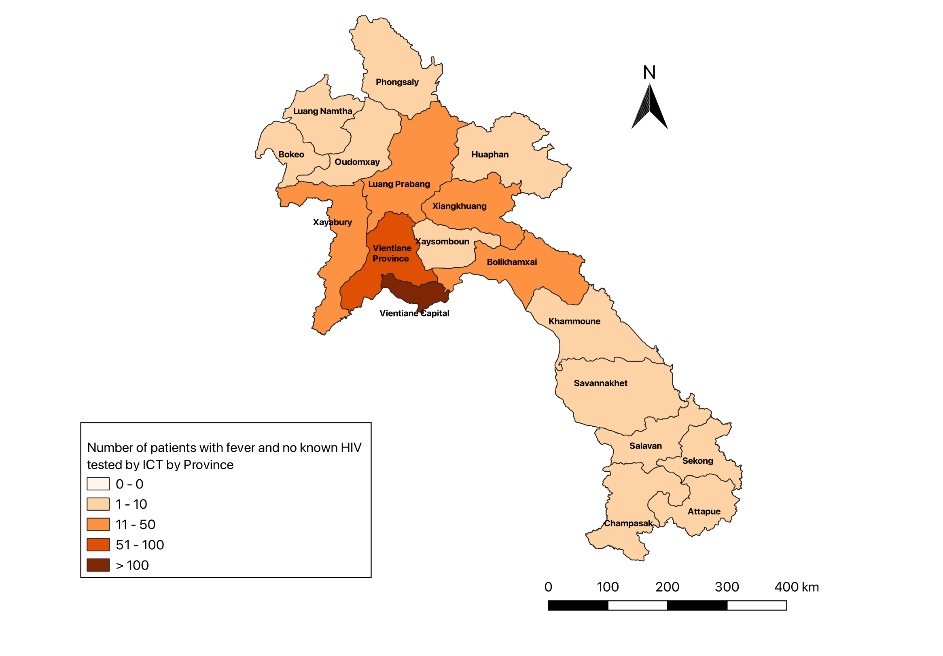


A

B

C

**Figure S2.** Province of origin for samples tested for *Leishmania.*A. Province location for people living with HIV tested by PCR. B. Province location for patients with fever and no known HIV tested by ICT. C. Province location of animals tested by ICT
